# Measuring bothersome menopausal symptoms: development and validation of the MenoScores questionnaire

**DOI:** 10.1186/s12955-018-0927-6

**Published:** 2018-05-16

**Authors:** Kamma Sundgaard Lund, Volkert Dirk Siersma, Karl Bang Christensen, Frans Boch Waldorff, John Brodersen

**Affiliations:** 1 Section of General Practice, Department of Public Health, University of Copenhagen, Øster Farimagsgade 5, Q, P.O. Box 2099, 1014 Copenhagen K, Denmark; 2 The Research Unit of General Practice, Department of Public Health, University of Copenhagen, Øster Farimagsgade 5, Q, 1014 Copenhagen K, Denmark; 3 Section of Biostatistics, Department of Public Health, University of Copenhagen, Øster Farimagsgade 5, Building 15 (15-2-12), P.O. Box 2099, DK-1014 Copenhagen K, Denmark; 4 The Research Unit for General Practice, Department of Public Health, University of Southern Denmark, J.B. Winsløws Vej 9, 5000 Odense C, Denmark; 5 Primary Health Care Research Unit, Region Zealand, 4180 Alleen 15, Sorø Denmark

**Keywords:** Questionnaire, Scale validation, Psychometrics, Item response theory, Rasch analysis, Patient-reported outcome measures, Menopause, Menopausal symptoms

## Abstract

**Background:**

The experience of menopausal symptoms is common and an adequate patient-reported outcome measure is crucial in studies where women are treated for these symptoms. The aims of this study were to identify a patient-reported outcome measure for bothersome menopausal symptoms and, in the absence of an adequate tool, to develop a new measure with high content validity, and to validate it using modern psychometric methods.

**Methods:**

The literature was reviewed for existing questionnaires and checklists for bothersome menopausal symptoms. Relevant items were extracted and subsequently tested in group interviews, single interviews, and pilot tests. A patient-reported outcome measure was drafted and completed by 1504 women. Data was collected and psychometrically validated using item-response theory Rasch Models.

**Results:**

All questionnaires identified in the literature lacked content validity regarding bothersome menopausal symptoms and none were validated using item-response theory. Our content validation resulted in a draft measurement encompassing 122 items across eight domains. Following psychometrical validation, the final version of our patient-reported outcome measure, named the MenoScores Questionnaire, encompassed 51 items, including one single item, covering 11 scales.

**Conclusion:**

Menopausal symptoms are multidimensional with some symptoms unquestionably related to the menopausal transition. We identified four constructs of importance: hot flushes, day-and-night sweats, general sweating, and menopausal-specific sleeping problems. The MenoScores Questionnaire is condition-specific with high content validity and adequate psychometrical properties. It is designed to measure bothersome menopausal symptoms and all scales are developed and psychometrically validated using item-response theory Rasch Models.

**Trial registration:**

Approved by the Danish Data Agency (J.nr. 2015–41-4057). Ethics Committee approval was not required.

**Electronic supplementary material:**

The online version of this article (10.1186/s12955-018-0927-6) contains supplementary material, which is available to authorized users.

## Background

Menopause is the cessation of women’s menstruation and can be determined retrospectively 12 months after the final menstrual period (FMP) [1, 2]. On average, women experience the menopausal transition in their mid-to-late forties [1] and the FMP in their early fifties, with large variations [1, 3, 4].

Around 75% of menopausal women experience hot flushes [5–7] and 10–20% of postmenopausal women find these symptoms very bothersome [5]. Some women also experience night sweats, emotional vulnerability, sleeping difficulties, fatigue, headache, joint and muscle pain, cognitive changes, vaginal dryness, and loss of sexual desire [1, 5, 8–10]. Menopausal symptoms are commonly experienced for 4–5 years in the years before and after the FMP; however, for some women the duration is longer [1, 6, 11].

Menopausal symptoms differ between cultures and ethnic groups, and also between individuals within a homogenous population [12, 13]. Therefore, measuring self-reported menopausal symptoms presents a challenge, and so does the distinction between menopausal symptoms and the symptoms of aging. Several questionnaires regarding menopausal symptoms exist. However, to help women who are bothered by menopausal symptoms it requires a PROM that focuses solely on the bothersome symptoms. Such a PROM must also possess high content validity as well as adequate psychometric properties. Item response theory Rasch models is preferred when establishing ideal measurement psychometric properties such as unidimensionality, invariance (specific objectivity or no differential item functioning), statistical sufficiency and additivity [14–16]. The aims of this study are threefold: 1) To review existing questionnaires and symptoms checklists (which we also refer to as questionnaires) measuring bothersome menopausal symptoms, and, if we cannot identify an adequate existing questionnaire from the literature search then: 2) To develop a patient-reported outcome measure (PROM) for bothersome menopausal symptoms with high content validity, and: 3) To validate this new PROM for dimensionality, invariance, known-groups validity, and reliability using modern psychometric methods.

## Methods

The study took place in Denmark and was divided into three phases: 1) a literature review; 2) qualitative interviews securing high face and content validity; 3) a validation survey where the draft PROM was distributed cross-sectionally and the data analyzed using classical test theory (CTT) and item response theory (IRT) models, securing high construct validity of the final PROM.

### Phase 1:Literature review

A literature search in PubMed, Embase, and the Cochrane Library was conducted at the end of 2014 and early 2015 to identify existing questionnaires encompassing menopausal symptoms. We also consulted gynaecologists and general practice specialists to locate relevant questionnaires. We included questionnaires that contained at least one item referring to a bothersome menopausal symptom. Questionnaires on the quality of life (i.e. no items referring to specific menopausal symptoms) or concerning interference with or reaction to menopausal symptoms were not included. Questionnaires had to be freely available and written in English, Swedish, Norwegian or Danish. To be interpreted as *adequate*, the identified questionnaires should have high content validity encompassing items that were up-to-date, not double-barrelled, or ambiguous. Moreover, the psychometric properties of the questionnaires should be assessed using IRT.

None of the identified questionnaires fulfilled all the above criteria. Therefore, we extracted an item-pool encompassing unique items about solely bothersome menopausal symptoms from the identified questionnaires. The meaningful content of relevant items was identified and assessed for redundancy, double-barrelled items were divided into separate items, and ambiguous items were rephrased. The items’ response options were not transferred [17]. The subject matter of these items was translated into Danish ad-hoc by KSL and JB. The unique items were grouped into domains by KSL based on clinical experience and the literature, and these were subsequently reviewed by JB. Any discrepancies were discussed until we reached consensus.

#### Phase 2: Qualitative interviews

To test the content validity (content relevance and content coverage) and the understandability of the unique items, two group interviews were conducted with women bothered by menopausal symptoms. The group interviews were audio-recorded, they lasted for two hours, and were moderated by KSL and JB. The first part of the interview was an open-ended discussion about bothersome menopausal symptoms. If new themes (suggested domains) were revealed in the discussion, we generated new items covering these themes using the women’s verbatim expressions from the audio recordings (see below). These new items were tested in the following group or in single interviews (see below). In the second part of the group interviews, the women were asked to assess if they found the subject matter of the unique items relevant. Items found irrelevant were deleted from the unique item-pool and, in case of lack of content coverage, new items were generated. We subsequently asked the women to which of the stated themes (suggested domain), their symptoms belonged. A draft PROM was created after the first group interview. At the end of the second group interview, the women were asked to complete the draft PROM. The themes (suggested domains), a recall period, and suggestions for response options were discussed. Instructions were tested for understandability.

Some symptoms postulated to be caused by menopause could also be caused by aging, therefore a global item was developed: “Have you, within the past three months, been bothered by menopausal symptoms?”, with four response options: “no, not at all”, “yes, a bit”, “yes, quite a bit”, or “yes, a lot”. Later, this global item was used to evaluate the association between women with and without bothersome menopausal symptoms and the scales’ ability to discriminate between the four groups in the global item: none, mild, moderate, or severe bothersome menopausal symptoms.

The draft PROM was further tested for functionality, understandability, and content validity in four single interviews conducted by KSL. The women included in these interviews were all bothered by menopausal symptoms. A paper version of the draft PROM was tested in the first two interviews and an online draft version was tested in the two final interviews. If any problems were revealed, they were corrected between interviews.

Finally, the online draft PROM was tested for functionality (including the response option) and understandability in four individual pilot tests, followed by a short interview, among women aged 50–64 where two of the women were bothered by menopausal symptoms.

The group and single interviews were audio-recorded and we measured the time taken to complete the PROM. Notes and important citations were listed during the interviews. After each interview the recording was audited by KSL and used when the key issues and results from the interviews were analyzed.

#### Phase 3: Validation survey and analysis

The final draft PROM was distributed by a link (SurveyXact) in emails, social media (Facebook groups for women), project research homepage [18], general practices, and the women’s lifestyle magazine “Liv” [19] (through their online newsletter and Facebook page). Women aged 45–65 years, with and without bothersome menopausal symptoms, were asked to complete the PROM.

##### Reliability and validity

To secure adequate psychometric properties of the final PROM we conducted Rasch analysis on the data collected verifying if items in each suggested domain fitted a partial credit Rasch model for polytomous items [20]. We tested differential item functioning (DIF) [21, 22], i.e. if items performed differently depending on the variables: occupation, education, living (living alone), smoking, BMI, age, hormonal intrauterine device, bilateral ovariectomized, hysterectomized, having menstruation within the past year. Local dependence (LD) was also evaluated [15, 23], i.e. whether items were correlated beyond what could be expected by measuring the same underlying construct using item screening and log-linear Rasch model tests [24, 25]. Where evidence of DIF and/or LD was disclosed, a log-linear Rasch model was considered indicating a scaling solution with desirable measurement properties [14]. Andersen’s conditional likelihood ratio test (CLR-χ^2^) was used to evaluate the overall model fit [26] and individual item fit was assessed by comparing observed and expected rank correlation between the item and rest-score (sum of other items in scale) [27]. Items that demonstrated the most problematic properties and/or poor fit were deleted stepwise from the scales, until fit of the Rasch model was achieved. Items with misfit but high face and content validity it were kept as a single item. Cronbach’s alpha was used as a measure of reliability [28, 29]. The Benjamini-Hochberg procedure was used to account for multiple testing [30].

The sum-scores of the resulting Rasch-fitting scales (see below) was tested by comparison to the global item. For each of the four categories of the global item the means and standard deviations (SD) of the sum-scores were calculated and compared using ANOVA; also, the order of the means in a sum-score should reflect the order of the categories of the global item. We calculated the number of subjects needed in a hypothetical randomized trial to find, with 80% power, the difference between the means corresponding to the two last categories of the global item in a t-test with a significance level of 5%; low numbers indicate a high discriminating ability. We used SAS v9.4 and DIGRAM v3.05.3 software.

The study was approved by the Danish Data Agency (J.nr. 2015–41-4057). Ethics Committee approval was not required.

## Results

### Phase 1

We identified 15 questionnaires written in English or Danish in the literature search, many of which referred to each other: Kupperman index [31, 32], Modified Blatt-Kupperman index [33, 34], Greene (1976) [35], Greene climacteric scale (GCS) [36], WHQ [37, 38], MENQOL [39], MENQOL-intervention [40], Menopause symptom list (MSL) [41], Menopause rating scale (MRS) [42], 10-items Cervantes scale (CS-10) [43], Menopause health state classification [44], Menopause health questionnaire [4], Neugarten and Kraines [45], Hvas et al. [46], MQOL [47]. None of the identified questionnaires were adequate in relation to *all* our adequacy criteria: some were not up-to-date [31, 35, 45], some not sufficiently validated [31, 32, 46] or with missing information about validation [4, 44]. Some had ambiguous or double-barrelled items [35, 36, 42], and some were primarily designed to measure quality of life in menopausal women [39, 40, 47] or economic evaluations of the impact of menopause [44], and not just the level of bothersome menopausal symptoms. None were assessed using IRT.

These questionnaires had in total 356 items, of which 126 were unique items divided into five domains (Additional file 1: Appendix 1).

### Phase 2

The first group interview included five women (aged 50–63 years), and the second included four women (aged 49–59 years).

In the two group interviews 95 (75.4%) of the 126 items were endorsed and 27 new items (five of these due to double-barrelled items) and three new domains were generated (Additional file 1: Appendix 1). In the first group interview it was revealed that hot flushes and day-and-night sweats were experienced as two different things (constructs). Some women were bothered by hot flushes but did not experience day-and-night sweats. Others were bothered by both hot flushes and day-and-night sweats, but described it as different experiences. This was confirmed in the second group interview.

The women agreed on a three-month recall period and preferred the four response options; “no, not at all”, “yes, a bit”, “yes, quite a bit”, or “yes, a lot” (Table 1. Item layout). In the sexual domain it was decided to create an additional response option “I do not know” for respondents not sexually active, with or without a partner. These preferences were later confirmed in the single interviews. By the end of the second group interview no new items or domains were generated.

**Table 1:** Example of item layout and response options

Have you – within the past three months – experienced the following symptoms?				
	No, not at all	Yes, a bit	Yes, quite a bit	Yes, a lot
I have had hot flushes during the day				
I have had hot flushes during the night				

Women interviewed individually were aged 50–52 and the women who participated in pilot testing were aged 50–64. In these interviews, almost all comments were about linguistic issues or layout suggestions and only one extra item was desired and another item perceived as redundant and deleted. At this point we achieved data saturation. Finally, one woman requested a comment box at the end of the PROM. Table 2 presents the number and age of participants in the interviews. The final version of the draft PROM encompassed 122 items covering 8 domains (Additional file 2: Appendix 2) and took, on average, 10 min to complete.

**Table 2:** Number and age of participants (BMS = bothersome menopausal symptoms)

	Number	Number with/without BMS	Age (women with BMS) Mean (range)	Age (women without BMS) Mean (range)
Group interviews	9	9/0	52.89 (49–63)	–
Single interviews	4	4/0	50.75 (50–52)	–
Pilot test	4	2/2	52 (50–54)	63 (62–64)
Survey (cross sectional)	1504	1073/431	51.97 (45–65)	50.69 (45–65)

### Phase 3

#### Survey

Within 48 h 1511 women had completed the draft PROM. Seven completed questionnaires were excluded; six respondents were under the age of 45 years and one respondent had ambiguous and inconsistent responses. The characteristics of the remaining 1504 respondents are listed in Tables 2 and 3.

**Table 3:** Characteristics of respondents (survey)

Characteristics/exogenous variables	Total no. (% of total)	Bothersome menopausal symptoms No. (%)
Total	1504 (100)	1073 (71.34)
Age (years):		
45–48	394 (26.20)	209 (53.05)
49–52	543 (36.10)	417 (76.80)
53–55	304 (20.21)	264 (86.84)
56–60	205 (13.63)	154 (75.12)
61–65	58 (3.86)	29 (50.00)
BMI		
0–18	10 (0.67)	8 (80.00)
19–24	682 (45.77)	469 (68.77)
25–29	518 (34.77)	379 (73.17)
≥ 30	280 (18.79)	206 (73.57)
Missing	14	
Occupation^a^		
Yes	1250 (83.11)	893 (71.44)
No	75 (4.99)	50 (66.67)
Sick leave	51 (3.39)	34 (66.67)
Retired or similar	128 (8.51)	96 (75.00)
Education		
No education	56 (3.73)	41 (73.21)
Skilled worker, apprentice, assistant nurse or likewise	273 (18.16)	206 (75.46)
Short higher education^b^ < 3 years	213 (14.17)	167 (78.40)
Medium higher education 3–4 years	633 (42.12)	449 (70.93)
Long higher education > 4 years	233 (15.50)	141 (60.52)
Other	90 (5.99)	63 (70.00)
Do not know	5 (0.33)	5 (100)
Missing	1	
Living alone		
Yes	310 (20.78)	214 (69.03)
No	1182 (79.22)	851 (72.00)
Missing	12	
Smoking		
Yes	238 (15.82)	179 (75.21)
No	1266 (84.18)	894 (70.62)
Gynecological history		
Hormonal intrauterine device		
Yes	247 (16.42)	144 (58.30)
No	1257 (83.58)	929 (73.91)
Bilateral ovariectomized		
Yes	40 (2.66)	32 (80.00)
No	1464 (97.34)	1041 (71.11)
Hysterectomized		
Yes	121 (8.05)	95 (78.51)
No	1383 (91.95)	978 (70.72)
Having menstruation within the past year		
Yes	717 (47.67)	464 (64.71)
No	787 (52.33)	609 (77.38)

^a^*Occupation* Employed, working or studying. ^b^higher education = education after high school or likewise

#### Psychometric analysis

The analyses revealed eleven uni-dimensional scales fitting a Rasch model. One single item was retained due to high face validity.

The final PROM was named the MenoScores Questionnaire (MSQ) and the eleven scales cover the constructs: hot flushes (HF), 2 items; day-and-night sweats (DNS), 2 items; general sweating (GS), 2 items; menopausal-specific sleeping problems (MSSP), 2 items; emotional (EM), 12 items; memory (MEM), 2 items; skin-hair (SH), 8 items; physical (PHY), 8 items; abdominal (ABD), 4 items; urinal-vaginal (URIN), 4 items, and sexual (SEX), 4 items. Including the retained single item (more tired than usual) the MSQ encompasses 51 items in total. Item numbers are listed in Table 4.

**Table 4:** Individual item fit

Item no.	Item wording	A priori domain	Final scale	Fit to Rasch model			Fit to log linear Rasch model		
				Observed	Expected	P	Observed	Expected	P
4	I have had hot flushes during the day	D2 - Vasomotor	HF	0.824	0.823	0.9107	0.821	0.820	0.9157
5	I have had hot flushes during the night	D2 - Vasomotor	HF	0.824	0.823	0.9107	0.821	0.820	0.9157
6	I have had bouts of sweating during the day	D2 - Vasomotor	DNS	0.694	0.691	0.8768	0.694	0.691	0.8757
7	I have had bouts of night sweats	D2 - Vasomotor	DNS	0.694	0.691	0.8768	0.694	0.691	0.8757
8	I have been sweating more than usual.	D2 - Vasomotor	GS	0.632	0.633	0.9720	0.632	0.633	0.9628
9	I have had cold sweats	D2 - Vasomotor	GS	0.632	0.633	0.9720	0.632	0.633	0.9628
10	I have not been able to sleep because of night sweats	D3 - Sleep	MSSP	0.872	0.870	0.8853	–	–	–
11	I have not been able to sleep because of hot flushes	D3 - Sleep	MSSP	0.872	0.870	0.8853	–	–	–
22	I have been depressed	D4 - Emotional	EM	0.717	0.680	0.0360	–	–	–
27	I have had mood swings	D4 - Emotional	EM	0.646	0.677	0.0545	–	–	–
30	I have felt anxiety	D4 - Emotional	EM	0.690	0.693	0.9022	–	–	–
31	I have felt nervous	D4 - Emotional	EM	0.696	0.680	0.3557	–	–	–
33	I have been needlessly worried	D4 - Emotional	EM	0.667	0.678	0.5050	–	–	–
34	I have been worried about having a nervous breakdown	D4 - Emotional	EM	0.727	0.700	0.1399	–	–	–
40	I have had less confidence	D4 - Emotional	EM	0.677	0.687	0.5561	–	–	–
43	I have not had the energy to socialize	D4 - Emotional	EM	0.671	0.677	0.7186	–	–	–
45	I have felt isolated	D4 - Emotional	EM	0.689	0.693	0.7989	–	–	–
47	I have done less than I would like	D4 - Emotional	EM	0.694	0.681	0.3996	–	–	–
48	I can accomplish less than I used to	D4 - Emotional	EM	0.680	0.682	0.9221	–	–	–
53	I have had difficulty concentrating	D4 - Emotional	EM	0.682	0.683	0.9548	–	–	–
54	My memory has been worse than usual	D4 - Emotional	MEM	0.923	0.923	0.9841	–	–	–
55	I have had problems with remembering everyday things	D4 - Emotional	MEM	0.923	0.923	0.9841	–	–	–
58	I have had dry skin	D5 - Skin, hair and mucosa	SH	0.462	0.411	0.0273	0.462	0.351	< 0.0001
62	I have had a crawling feeling over the skin	D5 - Skin, hair and mucosa	SH	0.411	0.418	0.8215	0.409	0.445	0.2133
63	I have had itching of the scalp	D5 - Skin, hair and mucosa	SH	0.436	0.405	0.2111	0.436	0.416	0.4045
64	I have had vaginal dryness	D5 - Skin, hair and mucosa	SH	0.418	0.431	0.5544	0.417	0.463	0.0378
65	I have had vaginal itching	D5 - Skin, hair and mucosa	SH	0.475	0.405	0.0131	0.474	0.478	0.8649
66	I have shed more hair than usual	D5 - Skin, hair and mucosa	SH	0.417	0.421	0.8541	0.417	0.418	0.9889
67	My nails split more than usual	D5 - Skin, hair and mucosa	SH	0.364	0.425	0.0128	0.371	0.423	0.0337
69	I have more body hair growth	D5 - Skin, hair and mucosa	SH	0.333	0.412	0.0062	0.333	0.346	0.6786
71	I have had heart palpitations	D6 - Physical	PHY	0.468	0.491	0.3039	0.468	0.491	0.3039
73	I have had headache	D6 - Physical	PHY	0.461	0.496	0.0934	0.461	0.496	0.0934
75	I have had a blind spot in front of my eye	D6 - Physical	PHY	0.510	0.510	0.9896	0.510	0.510	0.9896
76	I have been dizzy	D6 - Physical	PHY	0.533	0.485	0.0396	0.533	0.485	0.0396
80	One or more of my joints has been sore	D6 - Physical	PHY	0.528	0.513	0.4367	0.528	0.513	0.4367
84	I have had neck pain	D6 - Physical	PHY	0.530	0.518	0.5332	0.530	0.518	0.5332
86	I have had pins and needles in my feet	D6 - Physical	PHY	0.546	0.527	0.4502	0.546	0.527	0.4502
95	I have been more clumsy than usual	D6 - Physical	PHY	0.505	0.506	0.9606	0.505	0.506	0.9606
77	I have had nausea	D6 - Physical	ABD	0.421	0.464	0.1331	0.421	0.464	0.1331
96	My stomach has tended to be bloated	D6 - Physical	ABD	0.500	0.476	0.2837	0.500	0.476	0.2837
98	I have had uncontrollable loss of gas	D6 - Physical	ABD	0.507	0.472	0.1474	0.507	0.472	0.1474
102	My stool has been loose	D6 - Physical	ABD	0.453	0.466	0.6507	0.453	0.466	0.6507
106	I need to pass urine more frequently than usual	D6 - Physical	URIN	0.532	0.481	0.03241	0.532	0.492	0.0975
107	I sometimes leak urine	D6 - Physical	URIN	0.459	0.474	0.55320	0.459	0.489	0.2257
108	My urine has smelled different	D6 - Physical	URIN	0.511	0.482	0.28580	0.511	0.462	0.0770
110	My vaginal discharge has been different	D6 - Physical	URIN	0.419	0.483	0.03539	0.419	0.477	0.0569
115	I have had pain during intercourse	D7 - Sexual	SEX	0.701	0.629	0.0010	0.701	0.676	0.2232
116	I have had bleeding after intercourse	D7 - Sexual	SEX	0.714	0.699	0.5665	0.714	0.764	0.0222
117	I have been too tired for sex	D7 - Sexual	SEX	0.579	0.580	0.9703	0.579	0.550	0.1721
118	I have had difficulty achieving an orgasm	D7 - Sexual	SEX	0.535	0.576	0.0554	0.535	0.544	0.6740
91	I have felt more tired than usual	D6 - Physical	Single item	–	–	–	–	–	**–**

HF = hot flushes (2 items), DNS = day-and-night sweats (2 items), GS = general sweating (2 items), MSSP = menopausal-specific sleeping problems (2 items), EM = emotional (12 items), MEM = memory (2 items), SH = skin-hair (8 items), PHY = physical (8 items), ABD = abdominal (4 items), URIN = urin-vaginal (4 items), SEX = sexual (4 items)

#### Vasomotor symptoms

This suggested six-item domain showed misfit. Based on evidence of LD and results from the qualitative interviews, where hot flushes and day-and-night sweats were described as two different constructs, three two-item scales were formed. These scales all fitted a Rasch model and had no evidence of LD and were named: hot flushes (HF), day-and-night sweats (DNS) and general sweating (GS).

In the HF scale, item 4 (hot flushes during the day) showed DIF with respect to (wrt.) having menstruation within the past year (*p* = 0.0013), and item 5 (hot flushes during the night) showed DIF wrt. BMI (*p* = 0.0008). In the DNS scale, item 6 (sweats during the day) showed DIF wrt. BMI (*p* < 0.0001), and item 7 (night-sweats) showed DIF wrt. Having menstruation within the past year (*p* = 0.0045). In the GS scale there was no evidence of DIF.

#### Sleep

The suggested 10-item domain did not fit a Rasch model. A two-item menopausal-specific sleeping problems (MSSP) scale was found to fit a Rasch model with no evidence of DIF or LD.

#### Emotional

The suggested 36-item domain did not fit a Rasch model. We omitted poor fitting items and found a 12-item EM scale (items 22, 27, 30, 31, 33, 34, 40, 43, 45, 47, 48, 53) with adequate fit to the partial credit Rasch model, but with substantial evidence of LD. The LD suggests four clusters of items: depression (three items: 22 [been depressed], 27 [mood swings], 34 [worried about nervous breakdown]); anxiety (three items: 30 [felt anxiety], 31 [felt nervous], 33 [needlessly worried]); social (two items: 40 [less confidence], 45 [felt isolated]), and energy (four items: 43 [no energy to socialize], 47 [do less], 48 [can accomplish less] 53 [difficulty concentrating]). No satisfactory log-linear Rasch model could be identified.

We analyzed items 54 and 55 separately because of high content validity and because the content seemed different from the remaining items. They formed the Memory (MEM) scale where no DIF or LD was revealed.

#### Skin, hair and mucosa

This suggested 15-item domain did not fit a Rasch model, but an eight-item scale (58, 62, 63, 64, 65, 66, 67, 69), the skin-hair (SH) scale, was found to fit the log-linear Rasch model. Evidence of LD was disclosed for three item pairs: 62 (crawling feeling over the skin) and 63 (itching of the scalp); 64 (vaginal dryness) and 65 (vaginal itching); 66 (shed more hair than usual) and 67 (nails split more than usual). Item 62 showed DIF wrt. Smoking; item 64 showed DIF wrt. Age and wrt. Having menstruation within the past year, and item 65 showed DIF wrt. Having menstruation within the past year.

#### Physical

This suggested physical 41-item domain was divided into 3 hypothesized scales due to the content of the items: physical (PHY), 25 items; abdominal (ABD), 10 items, and urinary-vaginal (URIN), 6 items.

Physical (PHY) scale.

This 25-item scale was rejected, but a scale with eight items (71, 73, 75, 76, 80, 84, 86, 95) was found to fit the log-linear Rasch model where evidence of LD was found for the three item pairs 71 (heart palpitation) and 76 (been dizzy); 73 (headache) and 84 (neck pain); 80 (sore joints) and 86 (pins and needles in feet). Furthermore, item 73 showed DIF wrt. Age and item 80 showed DIF wrt. BMI.

#### Abdominal (ABD) scale

This 10-item scale was rejected, but a 4-item scale comprising the items 77, 96, 98, and 102 was found to fit a log-linear Rasch model. In this scale, LD was found between item 77 (nausea) and item 98 (uncontrollable loss of gas). Item 96 (bloated stomach) showed DIF wrt. Age and item 98 showed DIF wrt. Education.

#### Urinary-vaginal (URIN) scale

The 6-item scale was rejected, but a 4-item scale comprising the four items 106, 107, 108, and 110 was obtained. Item 108 (urine smells different) showed DIF wrt. Smoking and LD was found between item 106 (need to pass urine more frequently) and 107 (sometimes leak urine), and between 108 (urine smells different) and 110 (vaginal discharge has been different).

Item 91 (more tired than usual) did not fit any of the scales. The item was also tested with the MSSP scale but without a fit to a Rasch model. Finally, the item was tested with the three related items 92, 93, and 94 but they did not fit a Rasch model. Nevertheless item 91 was retained as a single item because of its high face validity.

#### Sexual

Four items (115, 116, 117, 118) from this domain fitted a Rasch model and were named the sexual (SEX) scale. LD was found between the items 115 (pain during intercourse) and 116 (bleeding after intercourse). Item 115 showed DIF wrt. Age and being bilaterally ovariectomized and item 116 showed DIF wrt. Having a hormonal intrauterine device and having menstruation within the past year; while item 117 (too tired for sex) showed DIF wrt. Living alone.

The SH, ABD scales showed signs of dichotomization in the category probability curves. The SH, ABD and SEX (with the additional response option “I do not know”) scales were re-tested in three single interviews (with women age 50 to 65) and all women preferred the three-response option instead of four (“no, not at all”, “yes, a bit”, or “yes, a lot”, plus the additional option in the SEX scale). In order to optimize model fit, the response options in these scales were reduced to the three options above (including the addition option in the SEX scale).

#### Work and spare time

Two-thirds of respondents were asked to complete this domain (i.e. women who claimed to be bothered by menopausal symptoms by answering “yes” to the global item). The 3-item domain fitted a Rasch model (*p* = 0.117) but items 1 and 3 with extremely poor item fit (*p* = 0.0001) and (p = < 0.0001). Thus, we decided to exclude this domain from the final PROM.

#### Menstruation

Only women who had menstruated within the past year were asked to complete this domain (approximately half of the respondents) (Table 3). This suggested 3-item domain did not fit a Rasch model (p = 0.000) and the items were not included in the final PROM.

#### Association (discrimination)

**Fig. 1:**
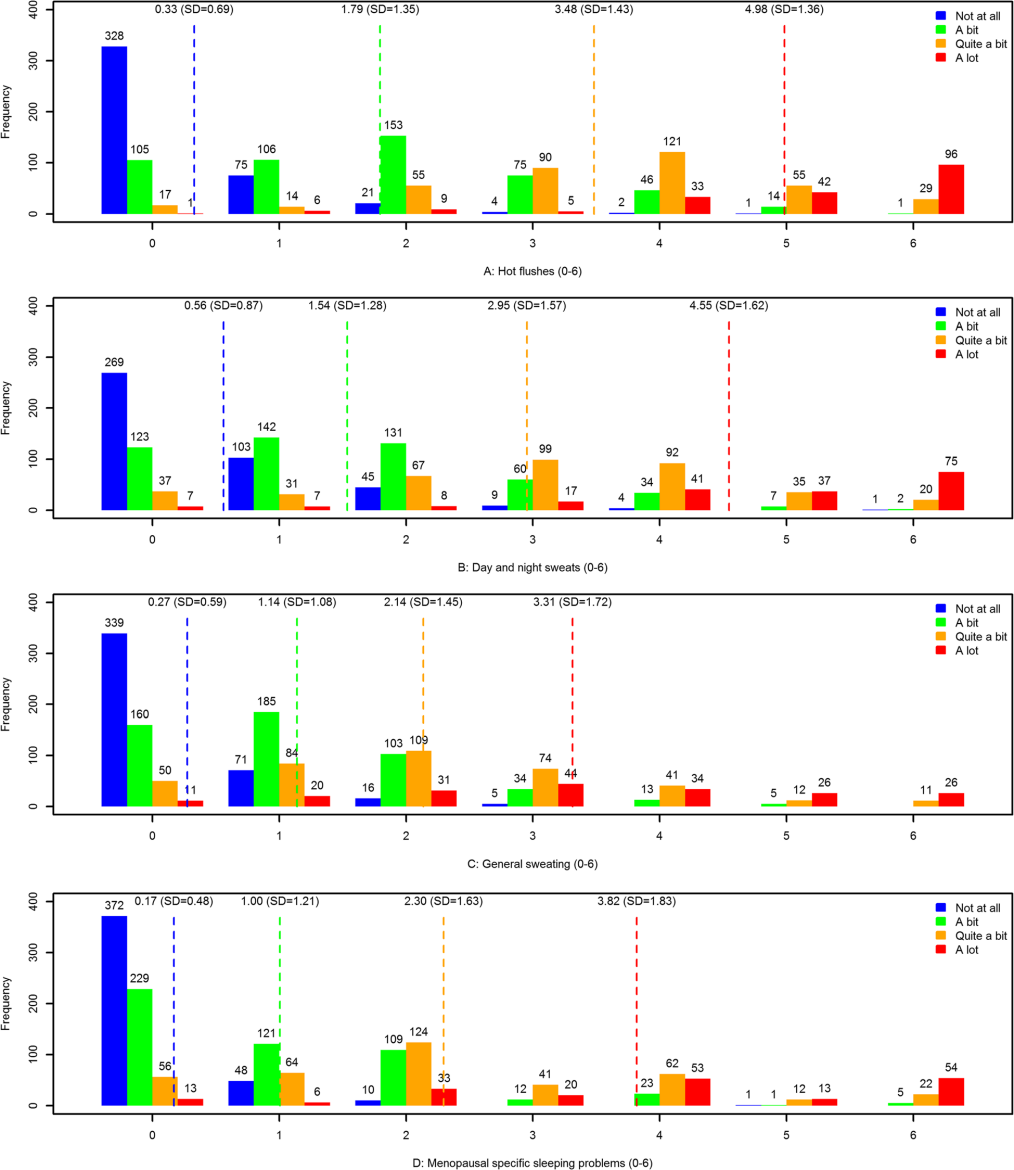
HF, DNS, GS, MSSP scales

The HF, DNS, GS, and MSSP scales showed best performance in discriminating between the response options of the global item (Fig. 1. HF, DNS, GS, MSSP scales). The discriminating ability is presented in Table 5.

**Table 5:** Fit statistics, the Cronbach’s alpha and discriminating ability of the scales included in the MSQ

Scale	Number of items	Fit to Rasch model			Fit to log linear Rasch model			Cronbach’s alpha	discriminating ability^a^
		χ^2^	df	P	χ^2^	df	P		
Vaso-motor	6	91.9	17	< 0.0001	–	–	–	0.90	
1. HF	2	6.1	5	0.2941	19.7	26	0.8057	0.85	30
2. DNS	2	9.9	5	0.0778	21.6	25	0.6588	0.76	34
3. GS	2	13.6	5	0.0183	13.2	17	0.7225	0.60	62
MSSP	10	308.2	29	< 0.0001	–	–	–	0.90	
4. MSSP	2	6.0	5	0.3077	–	–	–	0.87	42
Emotional	36	511.4	107	< 0.0001	–	–	–	0.97	
5. EM	12	45.1	35	0.1189	–	–	–	0.93	158
6. MEM	2	2.9	5	0.7176	–	–	–	0.90	258
SH	15	137.3	44	< 0.0001				0.81	
7. SH	8	27.7	23	0.2289	61.5	71	0.7828	0.71	300
Physical	25	768.6	74	< 0.0001				0.93	
8. PHY	8	25.1	23	0.3433	87.2	73	0.1235	0.79	218
Abdominal	10	219.0	29	< 0.0001				0.81	
9. ABD	4	13.2	11	0.2822	33.4	47	0.9260	0.63	522
Urinary	6	65.8	17	< 0.0001				0.74	
10. URIN	4	18.7	11	0.0674	7.0	32	1.0000	0.67	1040
Sexual	8	230.4	31	< 0.0001				0.95	
11. SEX	4	15.8	15	0.3981	37.6	58	0.9825	0.91	320

^a^Total number of responders needed to show a clinically relevant difference in symptoms (moderate vs. severe bothersome menopause-related symptoms) with an 80% power and 5% level of significance. Low numbers indicate a high discriminating ability.

df = Degrees of freedom, HF = hot flushes, DNS = day-and-night sweats, GS = general sweating, MSSP = menopausal-specific sleeping problems, EM = emotional, MEM = memory, SH = skin-hair, PHY = physical, ABD = abdominal, URIN = urin-vaginal, SEX = sexual

#### Reliability

The reliability of the scales was moderate to high with Cronbach’s alpha values between 0.60 and 0.91 (Table 5).

Table 4 presents individual item fit and Table 5 presents fit statistics, Cronbach’s alpha, and discriminating ability.

## Discussion

We found that all existing questionnaires lacked content validity regarding bothersome menopausal symptoms and none were validated using IRT. Moreover, they all regarded hot flushes and day-and-night sweats as a single construct, which this study could not confirm. We found that the suggested vasomotor domain was three-dimensional concluding that hot flushes and day-and-night sweats are two different constructs. This was revealed in the qualitative interviews and confirmed by the Rasch analysis. Furthermore, these findings were confirmed when screening potential participants for a current randomized controlled trial (RCT) [48]. This study also revealed that only some symptoms are unquestionably related to the menopausal transition and four constructs are of importance when measuring bothersome menopausal symptoms: hot flushes, day-and-night sweats, general sweating and menopausal-specific sleeping problems.

A strength of this study is the combination of rigorous qualitative and quantitative processes. Through the qualitative interviews we secured high content validity. Subsequently we used Rasch models to assess if the suggested domains behaved psychometrically as we expected. Another strength is the assessment of discriminating ability. Using the responses to the global item, in relation to the responses to the remaining items, we assessed how well the individual scales within the MSQ discriminated between the response options of the global item. We found the HF, DNS, GS and MSSP scales performed best in discriminating. Our interpretation of this is that only these constructs (HF, DNS, GS, MSSP) are unquestionably related to the menopausal transition. Many other symptoms may be, more or less, caused by aging.

A limitation could be that as the data was collected cross-sectionally, test-retest analysis is not reported. Women with bothersome menopausal symptoms report fluctuations in their symptoms from day-to-day. Therefore, a test-retest with a 2-week interval would not be meaningful. Instead we assessed the internal consistency of the scales using Cronbach’s alpha. A further limitation is the broad sampling procedure which makes it difficult to know exactly what population the sample is representative of, due to the element of self-selection inherent in survey data using web-based enrolment. The fact that Rasch validation is performed without distributional assumptions mitigates this challenge.

We identified DIF and LD in some of the final scales which may limit MSQ’s applicability in some situations. Items 4 and 5 from the HF scale and items 6 and 7 from the DNS scale all possessed DIF. Nevertheless, these items were maintained because of their high face validity. If the developed scales are used in a RCT, DIF is far less problematic because any exogenous variables will presumably be equally distributed among the randomized groups. However, if the scales are used in non-randomized studies, and any exogenous variables that can cause DIF appear in the studied cohort, one should adjust for the magnitude of the identified DIF [22]. Another approach would be to refrain from items possessing DIF or refrain from using the scales encompassing items possessing DIF [22].

Scales with many items may be preferred, since many items in a scale could increase the sensitivity, specificity, reliability, and ability to discriminate between the groups being tested. In the present study, our interest was to assess if the women were “not at all”, “a bit”, “quite a bit”, or “a lot” bothered by menopausal symptoms. We found the best discriminating scales among four 2-item scales: the vasomotor and sleeping scales (HF, DNS, GS and MSSP) and not among scales encompassing more items. There could be two reasons for this lack of discrimination: 1) LD, but even after deleting items with LD, these scales still did not discriminate as well as the scales from the vasomotor and sleeping domains; 2) that the subject matter of the other scales is related more to aging than to menopause.

Due to the large item-pool we identified, we could discharge problematic and poor fitting items using a stepwise procedure. However, we ensured that no important items were lost just because of a psychometric misfit. Therefore, items with high content validity but psychometric misfit were kept as a single item, e.g. item 91 (more tired than usual).

Even though the “work and spare time” domain fitted a Rasch model the items showed poor item fit. Since these items were not symptoms in themselves, but referred to how menopausal symptoms affected women’s work and spare time, we decided to disband these items and omit this domain from the final PROM. Moreover, the 3-item menstruation domain did not fit a Rasch model, and as these items were not of high relevance to this study, they were excluded from the final PROM.

Since the timing of menopause and the experience of menopausal symptoms vary so widely [1, 4], the MSQ is designed to measure self-reported bothersome menopausal symptoms both in peri- and post-menopausal women. The intention is for the MSQ to be used as an outcome measure in studies where women are treated for bothersome menopausal symptoms. The time needed to complete the MSQ is estimated at 5 min, as the MSQ contains fewer than half the items in the draft PROM.

The MSQ only addresses bothersome menopausal symptoms since these would be the target for treatment. It is important to note that some women also have positive experiences in relation to the menopause [49]; however, this is beyond the scope of the present study. The MSQ was developed in Danish and any new language or modified version may need an additional validation study to secure adequate measurement properties.

## Conclusion

Menopausal symptoms are multidimensional with only some symptoms unquestionably related to the menopausal transition. The MenoScores Questionnaire (MSQ) is a new, condition-specific PROM with high content validity and adequate psychometrical properties measuring bothersome menopausal symptoms. To the best of our knowledge this is the first PROM measuring only bothersome menopausal symptoms, wherein all scales are developed via interviews with women having bothersome menopausal symptoms and thereafter psychometrically validated using IRT Rasch Models. The focus on bothersome symptoms will assist with identifying and evaluating treatments for women bothered by menopausal symptoms.

## Additional files

Additional file 1: Appendix 1. Unique item-pool (separated into the suggested domains and the new items and domains generated in the interviews). (DOCX 28 kb)

Additional file 2: Appendix 2. Draft PROM. (DOCX 26 kb)

## Abbreviations

ABD: Abdominal
BMS: Bothersome menopausal symptoms
CCT: Classical test theory
Df: Degrees of freedom
DIF: Differential item functioning
DNS: Day-and-night sweats
EM: Emotional
FMP: Final menstrual period
GS: General sweating
HF: Hot flushes
IRT: Item response theory
LD: Local dependency
MEM: Memory
MSQ: MenoScores Questionnaire
MSSP: Menopausal-specific sleeping problems
PHY: Physical
PROM: Patient-reported outcome measure
RCT: Randomized controlled trial
SEX: Sexual
SH: Skin-hair
URIN: Urin-vaginal
Wrt: With respect to
