# Supplementary material for: Measuring bothersome menopausal symptoms: development and validation of the MenoScores questionnaire
Source: Health Qual Life Outcomes. 2018 May 16;16:97. doi: 10.1186/s12955-018-0927-6 (PMC5956969; doi:10.1186/s12955-018-0927-6)
Supplement: Supplementary file 1 — Appendix 1. Unique item-pool (separated into the suggested domains and the new items and domains generated in the interviews). (DOCX 28 kb) [file 12955_2018_927_MOESM1_ESM.docx]

**Appendix 1: Unique item-pool** (separated into the suggested domains and the new items and domains generated in the interviews).

1. **endorsed in the group interviews, b) endorsed and divided into two separate items after the group interviews, c) endorsed but rewording,** d) Not endorsed and excluded.

| **Vasomotor** | **Psychological** | **Physical/somatic** | **Sleep** | **Sexual** | **New items and domains after the group interviews** |
| --- | --- | --- | --- | --- | --- |
| 1. **Hot flushes^b^** *(split into two items, during day/night)* 2. **Night sweats^b^** *(an extra item generated; sweats during the day)* 3. **Cold sweats^a^** 4. **Involuntary sweating^c^** *(increased sweating)* 5. I find hot flushes embarrassing^d^ | 1. **Anxiety for no reason^a^** 2. Attacks of panic^d^ 3. **I feel sad^a^** 4. **Depressed^a^** 5. **More irritable than usual^a^** 6. **Feeling aggressive^a^** 7. **Feeling nervous^a^** 8. **Feeling tense^a^** 9. **Vulnerable^c^** 10. **Crying spells^a^** 11. **Worrying needlessly^a^** 12. **Mood swings^a^** 13. **Easily lose my temper^a^** 14. **Restless^a^** 15. **Excitable^a^** 16. **Lack of energy^a^** 17. **Difficulty in concentrating^a^** 18. **Poor memory^a^** 19. Forgetfulness^d^ 20. **Problem with remembering everyday things^a^** 21. **Difficulty doing my work because of my symptoms^a^** 22. **Mental exhaustion^a^** 23. **Lost interest in things^a^** 24. Anxious when I go out on my own^d^ 25. Feel life is not worth living^d^ 26. Dissatisfied with my personal life^d^ 27. **Accomplishing less than I used to^a^** 28. **Impatient ^a^** 29. **Wanting to be alone^a^** 30. Avoiding intimacy^d^ 31. Perception of being useless^d^ 32. Worry about growing old^d^ 33. Worry about missing work because of my symptoms^d^ 34. **Because of my symptoms I miss out leisure activities^a^** 35. More reclusive^d^ 36. **Feel isolated^a^** 37. **I do less than I would like^a^** 38. **Feel inadequate^a^** 39. **Because of my symptoms I sometimes have to get out of places^a^** 40. **Tearful^a^** 41. Worry about body^d^ 42. **Worry about nervous breakdown^a^** 43. Things I used to enjoy have become a bit of a chore^d^ | 1. **Heart palpitations^a^** 2. Heart discomfort^d^ 3. Heart tightness^d^ 4. **Breathing difficulties^a^** 5. **Headache^a^** 6. **More tired than usual^a^** 7. **dizzy^a^** 8. **Nauseous^a^** 9. My breasts feels uncomfortable^d^ 10. **Sore breasts^a^** 11. **Backache^a^** 12. **Muscle pain^a^** 13. **Joint pain^a^** 14. Pain in limbs^d^ 15. **Pain in back of neck^a^** 16. **Lower back pain^a^** 17. **I have pins and needles in my hands and feet^b^** 18. Body feels numb^d^ 19. **Body feels tingling^a^** 20. **Cold hands and feet^b^** 21. **Leg cramps^a^** 22. **Crawling feeling over the skin^a^** 23. **More clumsy than usual^a^** 24. **Stomach feels bloated^a^** 25. **Weight gain^a^** 26. **Uncontrollable loss of gas^a^** 27. **Uncontrollable loss of stool^a^** 28. **Flatulence^a^** 29. **Constipation^a^** 30. **Diarrhea^a^** 31. Abdominal cramps^d^ 32. **Good appetite^c^ *(increased)*** 33. Poor appetite^d^ 34. **Dry eyes^a^** 35. Change in skin appearance^d^ 36. **Greasy skin or acne^b^** 37. **Dry skin^a^** 38. **Need to pass urine/water more frequently^a^** 39. **Leak urine^a^** 40. Involuntary urination when laughing or coughing^d^ 41. Pain or burning when urinating^d^ 42. Difficulty in urinating^d^ 43. Bladder infections^d^ 44. **Vaginal dryness^a^** 45. **Vaginal itching^a^** 46. **Abnormal vaginal discharge^c^ (*different*)** 47. **Vaginal infections^a^** 48. Vaginal bleeding^d^ 49. **Vaginal spotting^a^** 50. **Irregular bleeding^a^** 51. **Heavy periods (menstruation)^a^** 52. **Physical exhaustion^a^** 53. **Undesirable body hair growth^a^** 54. **Facial hair growth^c^ (*beard growth*)** 55. **Decrease in physical strength^a^** 56. **Blind spot in front of eye^a^** 57. Feeling of suffocation^d^ 58. **Decrease in stamina^a^** 59. **Pressure/tightness in body^a^** 60. Pressure/tightness in head^d^ | 1. **Sleeplessness^c^** (*awake at night*) 2. **Difficulty in falling asleep^a^** 3. Wake early and sleep badly afterwards^d^ 4. **Wake up early^a^** 5. **Difficulty staying asleep^c^** (*sleeping through)* 6. **Night sweats keep me awake^a^** 7. **I take naps during the day^a^** | 1. **Change in sexual desire^c^ (*decrease*)** 2. **Change in sexual activity^c^ (*decrease*)** 3. Lost interest in sexual activity^d^ 4. **Change in sexual satisfaction^c^ (*decrease*)** 5. **Difficulty achieving orgasm^a^** 6. **Too tired for sex^a^** 7. **Because of vaginal dryness sexual intercourse has become uncomfortable^a^** 8. **Intercourse is painful^a^** 9. Pain inside during intercourse^d^ 10. **Bleeding after intercourse^a^** 11. My opportunity for sexual activity is limited^d^ | 1. **Hot flushes during night** 2. **Sweats during day** 3. **Breast tenderness** 4. **Pins and needles in my feet** 5. **Cold feet** 6. **Loose stool** 7. **Acne** 8. **Hot flushes keep me awake** 9. **Retention of body fluids** 10. **Nails split** 11. **Hangnails tear/spilt** 12. **Scalp itching** 13. **Altered urine odor** 14. **Altered odor in the groin area** 15. **Feeling less attractive** 16. **Dejected** 17. **Decreased confidence** 18. **Lacking initiative** 19. **Lacking energy to socialize** 20. **Less tolerant** 21. **Light sleep** 22. **Don’t feel rested when I wake up** 23. **Lying awake at night, thinking** 24. **More sensitive** 25. **More touchy** 26. **Shedding more hair** 27. **Itchy skin** 28. **Dry mucous membranes in the nose**   **3 new domains**   1. **Work and spare time** 2. **Skin-hair and mucosa** 3. **Menstruation** |
