# Supplementary material for: Measuring bothersome menopausal symptoms: development and validation of the MenoScores questionnaire
Source: Health Qual Life Outcomes. 2018 May 16;16:97. doi: 10.1186/s12955-018-0927-6 (PMC5956969; doi:10.1186/s12955-018-0927-6)
Supplement: Supplementary file 2 — Appendix 2. Draft PROM. (DOCX 26 kb) [file 12955_2018_927_MOESM2_ESM.docx]

**Appendix 2: Draft PROM**

| Item no. | Item | Suggested domain |
| --- | --- | --- |
| 1 | Difficulty to do my work or similar because I have been bothered by menopausal symptoms | D1 - Work/spare time |
| 2 | Because I have been bothered by menopausal symptoms I miss out leisure activities | D1 - Work/spare time |
| 3 | Because I have been bothered by menopausal symptoms I sometimes have to get out of places | D1 - Work/spare time |
| 4 | I have had hot flushes during the day | D2 - Vasomotor |
| 5 | I have had hot flushes during the night | D2 - Vasomotor |
| 6 | I have had bouts of sweating during the day | D2 - Vasomotor |
| 7 | I have had bouts of night sweats | D2 - Vasomotor |
| 8 | I have been sweating more than usual. | D2 - Vasomotor |
| 9 | I have had cold sweats | D2 - Vasomotor |
| 10 | I have not been able to sleep because of night sweats | D3 - Sleep |
| 11 | I have not been able to sleep because of hot flushes | D3 - Sleep |
| 12 | I have had trouble falling asleep | D3 - Sleep |
| 13 | I waken up too early in the morning | D3 - Sleep |
| 14 | I have needed a nap during the day | D3 - Sleep |
| 15 | I have slept more lightly | D3 - Sleep |
| 16 | I have had difficulty sleeping through | D3 - Sleep |
| 17 | I have been awake for a couple of hours at night | D3 - Sleep |
| 18 | I have been lying awake at night, thinking | D3 - Sleep |
| 19 | Though I slept through I do not feel rested | D3 - Sleep |
| 20 | I have been sad | D4 - Emotional |
| 21 | I have felt dejected | D4 - Emotional |
| 22 | I have been depressed | D4 - Emotional |
| 23 | I have been irritable | D4 - Emotional |
| 24 | I have been impatient | D4 - Emotional |
| 25 | I have felt aggressive | D4 - Emotional |
| 26 | I have lost my temper | D4 - Emotional |
| 27 | I have had mood swings | D4 - Emotional |
| 28 | I have been less tolerant | D4 - Emotional |
| 29 | I have been more touchy | D4 - Emotional |
| 30 | I have felt anxiety | D4 - Emotional |
| 31 | I have felt nervous | D4 - Emotional |
| 32 | I have felt tense | D4 - Emotional |
| 33 | I have been needlessly worried | D4 - Emotional |
| 34 | I have been worried about having a nervous breakdown | D4 - Emotional |
| 35 | I have had crying spells | D4 - Emotional |
| 36 | I have been tearful | D4 - Emotional |
| 37 | I have been more vulnerable | D4 - Emotional |
| 38 | I have been more sensitive | D4 - Emotional |
| 39 | I have felt less attractive | D4 - Emotional |
| 40 | I have had less confidence | D4 - Emotional |
| 41 | I have felt inadequate | D4 - Emotional |
| 42 | I have been lacking initiative | D4 - Emotional |
| 43 | I have not had energy to socialize | D4 - Emotional |
| 44 | I have wanted to be alone | D4 - Emotional |
| 45 | I have felt isolated | D4 - Emotional |
| 46 | I have lost interest in things | D4 - Emotional |
| 47 | I have done less than I would like | D4 - Emotional |
| 48 | I can accomplish less than I used to | D4 - Emotional |
| 49 | I have been restless | D4 - Emotional |
| 50 | I have been excitable | D4 - Emotional |
| 51 | I have been lacking energy | D4 - Emotional |
| 52 | I have felt mentally exhausted | D4 - Emotional |
| 53 | I have had difficulty in concentrating | D4 - Emotional |
| 54 | My memory has been worse than usual | D4 - Emotional |
| 55 | I have had problems with remembering everyday things | D4 - Emotional |
| 56 | I have had dry eyes | D5 - Skin, hair and mucosa |
| 57 | I have had dry mucous membranes in the nose | D5 - Skin, hair and mucosa |
| 58 | I have had dry skin | D5 - Skin, hair and mucosa |
| 59 | I have had greasy skin | D5 - Skin, hair and mucosa |
| 60 | I have had acne | D5 - Skin, hair and mucosa |
| 61 | I have had itchy skin | D5 - Skin, hair and mucosa |
| 62 | I have had a crawling feeling over the skin | D5 - Skin, hair and mucosa |
| 63 | I have had itching of the scalp | D5 - Skin, hair and mucosa |
| 64 | I have had vaginal dryness | D5 - Skin, hair and mucosa |
| 65 | I have had vaginal itching | D5 - Skin, hair and mucosa |
| 66 | I have shed more hair than usual | D5 - Skin, hair and mucosa |
| 67 | My nails spilt more than usual | D5 - Skin, hair and mucosa |
| 68 | My hangnails spilt more than usual | D5 - Skin, hair and mucosa |
| 69 | I have more body hair growth | D5 - Skin, hair and mucosa |
| 70 | I have more facial hair growth | D5 - Skin, hair and mucosa |
| 71 | I have had heart palpitations | D6 - Physical |
| 72 | I have had breathing difficulties | D6 - Physical |
| 73 | I have had headache | D6 - Physical |
| 74 | I have had a sensation of pressure in the body | D6 - Physical |
| 75 | I have had a blind spot in front of the eye | D6 - Physical |
| 76 | I have been dizzy | D6 - Physical |
| 77 | I have had nausea | D6 - Physical |
| 78 | My breasts have been tense | D6 - Physical |
| 79 | My breasts have been sore | D6 - Physical |
| 80 | One or more of my joints has been sore | D6 - Physical |
| 81 | I have had sore muscles | D6 - Physical |
| 82 | I have had back pain | D6 - Physical |
| 83 | I have had lower back pain | D6 - Physical |
| 84 | I have had neck pain | D6 - Physical |
| 85 | I have had pins and needles in my hands | D6 - Physical |
| 86 | I have had pins and needles in my feet | D6 - Physical |
| 87 | I have had pins and needles in the body | D6 - Physical |
| 88 | I have had cold hands | D6 - Physical |
| 89 | I have had cold feet | D6 - Physical |
| 90 | I have had leg cramps | D6 - Physical |
| 91 | I have felt more tired than usual | D6 - Physical |
| 92 | I have felt physical exhaustion | D6 - Physical |
| 93 | I have had decreased physical strength | D6 - Physical |
| 94 | I have had decreased stamina | D6 - Physical |
| 95 | I have been more clumsy than usual | D6 - Physical |
| 96 | My stomach has tended to be bloated | D6 - Physical |
| 97 | I have had flatulence | D6 - Physical |
| 98 | I have had uncontrollable loss of gas | D6 - Physical |
| 99 | I have had uncontrollable loss of stool | D6 - Physical |
| 100 | I have tended to be constipated | D6 - Physical |
| 101 | I have tended to have diarrhea | D6 - Physical |
| 102 | My stool has been looser | D6 - Physical |
| 103 | I have had increased appetite compared to usual | D6 - Physical |
| 104 | I have had a tendency to retain body fluids | D6 - Physical |
| 105 | I have gained weight | D6 - Physical |
| 106 | I need to pass urine more frequently than usual | D6 - Physical |
| 107 | I sometimes leak urine | D6 - Physical |
| 108 | My urine has smelled different | D6 - Physical |
| 109 | The odor from my groin area has changed | D6 - Physical |
| 110 | My vaginal discharge has been different | D6 - Physical |
| 111 | I have had vaginal infections | D6 - Physical |
| 112 | I have had decreased sexual desire | D7 - Sexual |
| 113 | I have had decreased sexual activity | D7 - Sexual |
| 114 | Because of vaginal dryness sex has become uncomfortable | D7 - Sexual |
| 115 | I have had pain during intercourse | D7 - Sexual |
| 116 | I have had bleeding after intercourse | D7 - Sexual |
| 117 | I have been too tired for sex | D7 - Sexual |
| 118 | I have had difficulty achieving an orgasm | D7 - Sexual |
| 119 | I have had decreased sexual satisfaction | D7 - Sexual |
| 120 | I have had vaginal spotting | D8 - Menstruation |
| 121 | I have irregular bleeding | D8 - Menstruation |
| 122 | I have had heavy bleeding periods | D8 - Menstruation |
